# From Cup to Scan: The Impact of Black Tea on Magnetic Resonance Cholangiopancreatography Signal Suppression

**DOI:** 10.2174/0115734056393392250414074014

**Published:** 2025-04-17

**Authors:** Sihua Liang, Yiman Wang, Huiyi Liang, Xuefen Yu, Nengwei Wang, Lin Qiu

**Affiliations:** 1 Medical Imaging Center, The First Affiliated Hospital of Jinan University, Guangzhou 510630, Guangdong, China; 2 Department of Ultrasound, The First Affiliated Hospital of Jinan University, Guangzhou 510630, Guangdong, China; 3 Department of General Medicine, The First Affiliated Hospital of Jinan University, Guangzhou 510630, Guangdong, China

**Keywords:** Magnetic Resonance Cholangiopancreatography, Contrast Agents, Gastrointestinal Tract, Black Tea

## Abstract

**Aims:**

The aim of this study is to evaluate the potential of black tea as a negative oral contrast agent in Magnetic Resonance Cholangiopancreatography (MRCP) to improve image quality by reducing gastrointestinal fluid signals.

**Background:**

Retained gastrointestinal fluids can interfere with ductal imaging during MRCP, and suitable oral negative contrast agents are not widely available.

**Method:**

Two types of black tea (Lapsang Souchong and Yinghong NO9) were tested in vitro at different concentrations (3g, 6g, and 9g) to assess their T2 signal suppression. The tea with the best signal suppression was selected for a prospective clinical study involving 51 patients undergoing MRCP. Signal intensity, signal-to-noise ratio (SNR), and contrast-to-noise ratio (CNR) were measured before and after black tea administration.

**Result:**

*In vitro* experiments showed that the 9g concentration of Lapsang Souchong tea provided the most effective T2 signal suppression, with manganese and iron ion concentrations of 4.705 mg/L and 0.040 mg/L, respectively. In the clinical study, paired T-tests revealed a significant decrease in gastrointestinal fluid signals after black tea administration, with a mean signal intensity reduction in the stomach and duodenum. The SNR in the duodenal bulb increased significantly, while no significant differences were observed in SNR and CNR in other gastrointestinal segments.

**Conclusion:**

Black tea, rich in iron and manganese, effectively reduces gastrointestinal fluid signals, potentially enhancing MRCP image quality. Further research is warranted to explore its clinical application.

## 1 INTRODUCTION

Magnetic Resonance Cholangiopancreatography (MRCP) is a non-invasive imaging modality that provides high-resolution visualization of the biliary and pancreatic ducts, rendering it particularly effective for diagnosing conditions such as choledocholithiasis, strictures, and other biliary pathologies [1-3]. Despite extensive pre-examination prepa-ration, fluid retention in the gastrointestinal tract persists, significantly obstructing the clear visualization of the anatomical structures of the pancreas and biliary ducts. Although advanced imaging sequences, such as compressed sensing and gradient-and spin-echo, or deep learning reconstruction techniques improve significantly the signal-to-noise ratio (SNR) and contrast-to-noise ratio (CNR) [4-6]. However, these methods are still difficult to completely eliminate the interference of fluid signals in the gastrointestinal tract. How to effectively inhibit the interference of fluid signals in the digestive tract remains an urgent problem to be addressed.

Patient critical nursing step preparation for MRCP is essential for optimal image quality. In previous studies, numerous scholars have attempted to enhance MRCP image quality using various gastrointestinal negative contrast agents, including oral agents like gadopentetate dimeglumine and superparamagnetic iron oxide [7-9]. However, these metallic drugs often have poor palatability, making them difficult for patients to accept, and they can lead to gastrointestinal symptoms. Some fruit juices, such as pineapple and blueberry juice, can effectively reduce the T2 signal of fluids [10-13]. But, they carry the potential risks of allergies or increased blood glucose levels, and the concentration of paramagnetic substances is often low and difficult to control [11, 14]. Black tea is a readily available beverage rich in paramagnetic substances, primarily iron and manganese ions, which have been studied for use as a negative contrast agent in preparation for MRCP examinations [15]. Due to variations in type, region of growth, and processing techniques, there are differences in trace elements within the tea [16, 17], which may lead to varying effects on T2 signal suppression, necessitating further research.

In this study, we selected two widely available black teas and brewed them at different ratios for in vitro testing. We analyzed the effect of these samples on MRCP sequence signal suppression and quantified the iron and manganese ion content. The most effective concentration was then used in a randomized patient trial to assess the impact of oral black tea on MRCP image quality. The purpose is to eliminate the interference of liquid signals in the gastrointestinal tract, using black tea as a negative oral contrast agent in search of a safe, convenient, and rapid preparation protocol for MRCP examination.

## 2 METHODS

This study was approved by the ethics committee of the first affiliated hospital of Jinan University, and all patients signed informed consent.

### 2.1 *In vitro* Assays and Data Analysis

In this study, we selected two prevalent black teas for our investigation: Lapsang Souchong from Fujian Province, China, and the Yinghong No. 9 cultivar from the Research Institute of Guangdong Academy of Agricultural Sciences, China. These teas were prepared in 3g, 6g, and 9g groups using 200mL of distilled water heated to 100°C, and steeped for exactly 5 minutes without stirring, after which the tea leaves were immediately removed. Once the temperature dropped to around 50°C, the infusion was transferred into 10ml test tubes. Control test tubes were filled with pure water at the same temperature. Both sets of test tubes were then subjected to image acquisition using a 1.5 T MR scanner (Signa HDxt 1.5T; GE Healthcare, Chicago, IL, USA); a body coil and a 16-channel abdomen phased-array coil were used. 3D-MRCP was performed using fast recovery fast spin echo (FRFSE) in the coronal plane. The parameters were as follows: TR/TE 4000-7500/730-1000 ms, slice thickness 1.6 mm, no gap between sections, matrix 288×256, FOV 36-40 cm, bandwidth 41.76 Hz, flip angle 90°, number of excitations (NEX)1, Phase encoding direction right-left (RL), frequency encoding direction superior-inferior (SI), echo train length 70 and imaging options: RT, ZIP512, ZIP2 were selected. Fat saturation was employed to suppress interference from the surrounding fat tissues. After scanning, the samples were dispatched to the Jinan University's testing center for quantitative determination of manganese and iron ions using Inductively Coupled Plasma-Atomic Emission Spectrometry (ICP-AES).

The acquired images were transferred to a postprocessing workstation (Workstation version 4.7 image postprocessing workstation, GE Healthcare) for image analysis. Two MR technologists (Yiman Wang and Nengwei Wang, with 4 years and 12 years of experience in MR operation, respectively) manually delineated regions of interest (ROIs). Each technologists delineated three ROIs on the median axial plane on consecutive slices for measurements and calculated the average. The average of the values obtained by the two technologists was taken as the final measurement, during the delineation of ROIs. The ROI size was 50 mm^2^ and the ROIs were selected within the slice with the largest area whenever possible. The signal intensity and standard deviation within the samples, as well as the signal intensity and standard deviation of the background, were measured respectively. Signal-to-Noise Ratio (SNR) and Contrast-to-Noise Ratio (CNR) were calculated. The formula for calculating SNR was: [mean of SI in each region]/[standard deviation of air SI]. The formula for calculating CNR was [mean of SI in each region - mean of SI in adjacent tissue]/[standard deviation of air SI] [15].

### 2.2 Clinical Application and Image Analysis

Through the analysis of the samples, we identified the optimal concentration for suppressing signals within fluids. Prospectively, we enrolled patients who underwent MRCP examinations at our hospital from November 2023 to November 2024. The inclusion criteria were as follows: 1. Patients with clinical diagnoses of biliary system diseases suitable for MRCP; 2. Patients capable of cooperating with breath-holding and breath control; 3. Patients who fasted and refrained from drinking for 6-8 hours prior to the examination but still had residual fluids in the gastrointestinal tract that interfered with the imaging of the pancreatobiliary system; 4. All patients provided informed consent. The exclusion criteria included: 1. Patients with contraindications for MR imaging; 2. Patients with intestinal obstruction, acute pancreatitis, or ascites; 3. Patients with gastrointestinal diseases not suitable for tea consumption, such as esophageal inflammation, gastroesophageal reflux, or peptic ulcers; 4. Patients with hearing impairments who could not cooperate with the examination; 5. Other causes of artifacts that interfered with image quality such as internal fixations from lumbar spine surgery or postoperative bowel movement artifacts. Eligible patients who met the inclusion criteria were administered 200 mL of black tea orally immediately after completing the initial MRCP examination, with the second MRCP scan performed precisely 5 minutes after tea consumption.

In this study, a comparative image quality assessment was conducted on two MRCP imaging sessions. Two radiologists, Huiyi Liang, with 4 years and Lin Qiu, with 15 years of experience in abdominal imaging, manually delineated ROIs on 3D MRCP images.

They measured the signal values in the stomach and duodenum before and after black tea consumption, as well as background signal values. The size of the ROIs, adjusted to 100-300 mm^2^ based on the anatomical structures, was kept within the contours of the stomach or duodenum while avoiding surrounding cystic lesions, as shown in Fig. (**1**). Each area was outlined three times to calculate an average value, and the final measurement was the average of the measurements from both radiologists. SNR and CNR were calculated using the afore mentioned formulas. Additionally, they evaluated the obstruction of the pancreatic and bile ducts and the structures interfering with the pancreatic and bile ducts.

### 2.3 Statistical Analysis

Sample size calculations were performed using PASS software (version 15.0.3; NCSS, UT, USA), a comprehensive tool for determining the number of subjects required to achieve the desired statistical power and significance level in clinical studies. Given the absence of direct literature evidence supporting the magnitude of signal reduction in the gastrointestinal tract following the ingestion of black tea, we relied on in vitro experimental predictions. It was estimated that a signal reduction of approximately 500 would be visually discernible, with a standard deviation of 700. With a significance level (α) set at 0.05 and a power of 0.9 (1-β), the software calculated that a sample size of 25 cases was necessary to detect a significant difference. Anticipating a dropout rate of 20%, the final required sample size was adjusted to 32 cases to account for potential attrition during the study. This rigorous approach to sample size estimation ensures that our study is adequately powered to detect a clinically significant difference in MRCP image quality following the ingestion of black tea.

Statistical analysis was performed using SPSS version 25.0 and MedCalc statistical software version 20.2. Interobserver agreements for the signal values measurements were calculated by the intraclass correlation coefficients (ICCs) and 95% confidence intervals. The agreement was interpreted according to interobserver criteria as poor (ICC < 0.21), discrete (ICC: 0.21–0.40), moderate (ICC: 0.41–0.60), good (ICC: 0.61–0.80), and excellent (ICC > 0.80). We illustrated interobserver agreement using Bland-Altman analysis. To assess the differences in MRCP sequence signal values, SNR, and CNR across various samples and concentrations, we employed one-way ANOVA, a standard approach for comparing means among three or more independent groups. For evaluating the changes in signal values, SNR, and CNR within the gastrointestinal tract before and after black tea ingestion in the same patients, paired T-tests were used, which is appropriate for comparing two related samples. A p-value threshold of less than 0.05 was established to indicate statistical significance, aligning with common practices in the field to denote a result as statistically meaningful.

## 3 RESULTS

In this study, we evaluated the signal suppression effects of various concentrations of two black teas on MRCP images and identified the optimal preparation as 9g of Lapsang Souchong steeped in 200ml of boiling water for 5 minutes, yielding manganese and iron ion concentrations of 4.705mg/L and 0.040mg/L, respectively. A total of 53 patients were enrolled; however, two patients were excluded due to respiratory motion artifacts, resulting in a final analysis of 51 patient datasets. Image analysis revealed that the common bile duct's mid to lower segment was most susceptible to interference, primarily from fluid in the duodenal bulb, as shown in Table **1**.

In both *in vitro* experiments and observational studies involving patients, the inter-observer reliability was found to be excellent, with ICCs of 0.996 (95% confidence interval [CI] 0.995 to 0.997) and 0.998 (95% CI 0.998 to 0.998), respectively. Bland-Altman analyses confirmed a strong inter-rater agreement for MR signal measurements in the in vitro MRCP sequences, with a mean difference of -2.5% and limits of agreement between -38.9% and 34.0%. Similarly, in the clinical testing segment, the assessment of MR signals in the stomach and duodenum from MRCP sequences showed substantial inter-rater reliability, with a mean difference of -2.3% and limits of agreement from -23.3% to 18.7%, as depicted in Fig. (**2**).

*In vitro* experiments demonstrated that both black tea varieties effectively reduced the signal of fluids, with signal intensity in the MRCP images decreasing as tea concentration increased. Concurrently, the concentration of iron and manganese ions in the liquids increased, as shown in Fig. (**3**). Notably, the 9g concentration of Lapsang Souchong exhibited the lowest signal, as depicted. Statistical analysis revealed significant differences in the impact of various concentrations of Yinghong No. 9 and Lapsang Souchong on MRCP sequence signal values, as detailed in Table **2**.

Following tea ingestion, a noticeable reduction in the signal from gastrointestinal fluids was observed, enabling the visualization of previously obscured pancreatobiliary structures, as depicted in Fig. (**4**). Paired T-test analysis revealed that the signal intensity and contrast-to-noise ratio in the gastrointestinal tract were significantly decreased after tea consumption, with statistical significance, as shown in Table **3**. Additionally, the signal-to-noise ratio in the descending portion of the duodenum increased significantly. In contrast, no significant statistical differences were found in the signal-to-noise ratios of other gastrointestinal segments.

## 4 DISCUSSION

In this investigation, we utilized an in vitro methodology to ascertain the optimal dosage of black tea for attenuating T2-weighted MR signals within the gastrointestinal tract. Our results demonstrated that a 9g infusion of Lapsang Souchong tea exhibited the most pronounced suppressive effect on T2-weighted signals concurrent with elevated levels of manganese and iron ions. Thereafter, we amassed data from a patient cohort comprising 51 individuals for comprehensive analysis. Comparative assessments of MRCP images pre- and post-tea ingestion indicated that this specific concentration significantly reduced fluid-related signals in the gastric and duodenal regions, consequently augmenting the delineation of the entire pancreatobiliary tract.

Lesions in the common bile duct typically lead to low-level biliary obstruction [18-20]. The principal aim of pancreaticobiliary imaging is to pinpoint the location of the lesion, which typically results in localized biliary stenosis and upstream dilation of the pancreaticobiliary system [21]. Our statistical findings indicate that biliary obstruction predominantly occurs in the mid-lower segment of the common bile duct, a region particularly susceptible to interference from fluids within the duodenal bulb and descending segment [22]. Although many negative contrast agents can reduce the signal of gastrointestinal tract fluids, a recent review study reported that few studies have conducted a detailed analysis of the specific pancreaticobiliary sites susceptible to such interference [23]. In this study, by comparing MRCP images before and after the ingestion of black tea, we discovered that the fluid signals within the gastrointestinal tract were well suppressed, leading to better visualization of the middle and lower segments of the common bile duct, the pancreaticobiliary ampulla, and the major duodenal papilla. Moreover, the image quality of the duodenal bulb was significantly improved after black tea ingestion, likely due to local anatomical factors that reduced background noise and thus enhanced the signal-to-noise ratio.

Our study aligns with the majority of scholarly research, indicating that black tea can reduce signals in the gastrointestinal tract of MRCP sequences primarily due to its rich content of iron, manganese, and magnesium ions, which are paramagnetic substances that shorten T2 relaxation times and thereby suppress T2 signals [24]. Previous studies have seldom investigated the variety, concentration, and brewing methods of black tea, nor have they quantified the iron and manganese ions within it [15, 25]. Due to variations in trace element content associated with different types and regions of growth of black tea [26], in this work, we observed that with increasing tea leaf content, the concentrations of iron and manganese ions in both types of black tea increased over a certain brewing period, leading to a progressive enhancement in the suppression of T2 signals. Specifically, we compared the effects of different qualities of Lapsang Souchong and Yinghong No. 9 black tea brewed in 200ml of boiling water for 5 minutes. We found that 9g of Lapsang Souchong black tea provided the best liquid T2 signal suppression effect, with the highest concentrations of manganese and iron ions. This indicates that the concentrations of manganese and iron ions are crucial for the effectiveness of black tea as a negative contrast agent in MRCP imaging.

This study presents a straightforward method to reduce fluid signals within the gastrointestinal tract during MRCP examinations. However, there are several limitations that warrant mention. Firstly, the variety of black teas available on the market is extensive, and the content of iron and manganese ions may vary among teas grown in different geographical environments, harvest years, seasons, leaf surfaces, leaf size, and processed using different methods. Our study only included two common types of black tea, and other varieties were not investigated. Secondly, we only measured the content of iron and manganese ions; other elements that could potentially reduce T2 signals were not assessed. Lastly, the amount of black tea consumed by patients during MRCP examinations was fixed, but due to the inability to accurately measure the amount of residual fluids, the diluted iron ion concentration after ingestion was inconsistent, and further analysis on the effect of signal reduction was not conducted. Despite these limitations, our findings contribute to the understanding of how black tea can be utilized to improve MRCP image quality by reducing gastrointestinal fluid signals.

## CONCLUSION

In summary, our study demonstrates that black tea serves as an effective negative oral contrast agent by significantly suppressing T2-weighted MR signals in the gastrointestinal tract. This suppression enhances the visualization of pancreatic and biliary ducts, particularly in regions where gastrointestinal overlap typically obscures imaging. The efficacy of black tea is attributed to its iron and manganese ion content, which contribute to T2 signal reduction. This simple, safe, and widely accessible method offers a practical solution for improving MRCP image quality by minimizing gastrointestinal fluid interference. Further research should explore the potential of other tea varieties and refine the protocol to optimize its clinical application.

## Figures and Tables

**Fig. (1):**
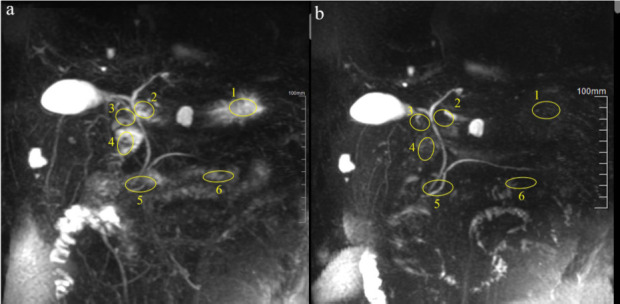
MRCP images before (**a**) and after (**b**) oral consumption of black tea, with ROIs selected for the gastric body (1), antral region (2), duodenal bulb (3), descending part of the duodenum (4), horizontal part of the duodenum (5), and ascending part of the duodenum or small intestine (6) to measure signal values.

**Fig. (2):**
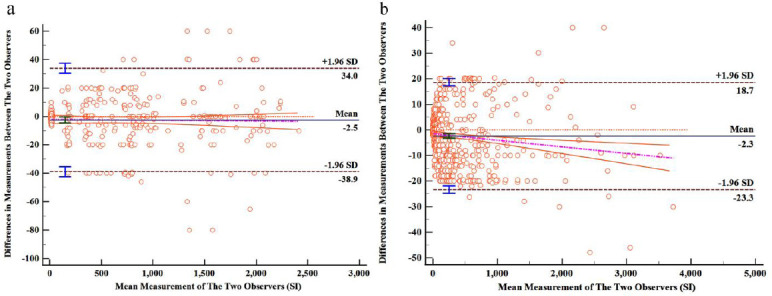
Bland–Altman analyses. The Bland–Altman analysis of the measurements of different samples by two observers in vitro showed a high consistency in interobserver reliability (**a**). The Bland–Altman analysis of the measurements of various gastrointestinal indicators by two observers showed a high consistency in interobserver reliability (**b**).

**Fig. (3):**
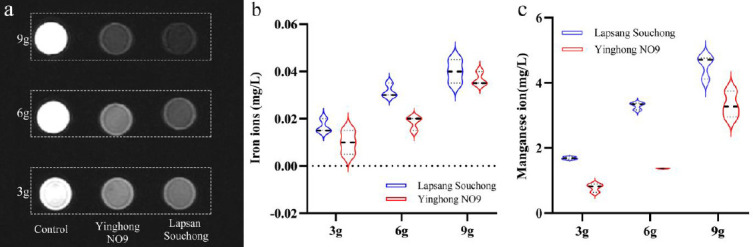
MRCP signal images of different concentration samples (**a**), with 9g of Lapsang Souchong black tea showing the lowest signal; the concentration of iron (**b**) and manganese (**c**) in different concentration samples, where Lapsang Souchong black tea contains higher levels of iron and manganese compared to Yinghong NO9.

**Fig. (4):**
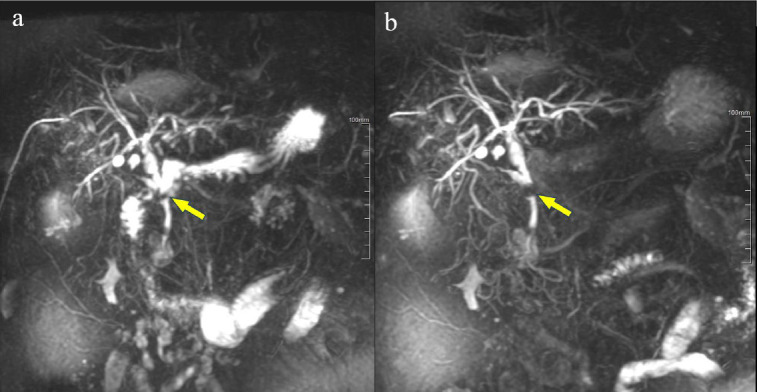
Male, 57 years old, obstructive jaundice, post-biliary drainage via percutaneous transhepatic cholangiography. (**a**) Dilated common bile duct with significant fluid signal interference in the gastric antrum (arrow), incomplete visualization of the common bile duct. (**b**) After oral consumption of black tea, suppression of signals in the gastric antrum and duodenum, revealing the obstructed part of the common bile duct (arrow).

**Table 1:** Clinical data and demographic data of the patients.

**Characteristic**	**Value (n=51 patients)**
**Age**	52(19~75)
**Gender**	
Male	38(74.5%)
Female	13(25.5%)
**Biliary obstruction**	
With	32(62.7%)
Without	19(37.3%)
**Covered segment**	
Left/right hepatic ducts	5(8.6%)
Common hepatic duct	15(25.8%)
Gallbladder and cystic duct	27(46.6%)
Common bile duct	31(53.4%)
Main pancreatic duct	15(25.9%)
**Adjacent organ segment**	
Body of the stomach	6(10.3%)
Pyloric antrum	27(46.6%)
Duodenal bulb	25(43.1%)
Descending part of the duodenum	21(36.2%)
Horizontal part of the duodenum	8(13.8%)
Ascending part of the duodenum and jejunum	13(22.4%)

**Table 2:** Comparison of images of the two black teas with different concentrations and controls in MRCP sequences.

-	-	3g	6g	9g	*F*	*p*
Signal intensity	Lapsang Souchong	701.859±100.418	456.400±74.660	175.377±17.533	339.066	<0.001
	Yinghong NO9	900.904±82.367	750.189±86.529	370.843±21.617	394.779	<0.001
	Control*	1637.404±410.619	1628.452±333.672	1720.561±299.122	0.545	0.582
	*F*	82.487	184.777	749.773	-	-
	*p*	<0.001	<0.001	<0.001	-	-
SNR	Lapsang Souchong	4.016±0.699	4.339±0.789	3.635±0.401	7.618	0.001
	Yinghong NO9	4.129±0.844	4.464±1.006	4.392±0.493	1.233	0.297
	Control*	3.182±0.656	3.120±0.786	3.152±0.635	0.051	0.95
	*F*	12.803	19.805	37.763	-	-
	*p*	<0.001	<0.001	<0.001	-	-
CNR	Lapsang Souchong	106.953±17.724	68.820±13.583	24.884±3.393	257.879	<0.001
	Yinghong NO9	138.100±17.310	114.913±19.257	55.368±5.879	201.493	<0.001
	Control*	252.372±68.154	250.198±51.995	264.599±47.007	0.491	0.614
	*F*	71.507	177.151	723.166	-	-
	*p*	<0.001	<0.001	<0.001	-	-

*The control group uses distilled water, with no concentration differences.

**Table 3:** Results of paired T-tests for observed indicators in various parts of the gastrointestinal tract before and after oral consumption of black tea.

Anatomical Site	Indicators of observation	Before Administration	After Administration	95% Confidence Interval		*t*	*P*
Lower	Upper
Stomach body	Signal intensity	135.260(65.570~729.330)	39.320(18.140~357.770)	87.773	441.702	3.005	0.004
	SNR	2.436(1.774~2.949)	3.443(2.090~4.890)	-2.085	2.223	0.064	0.949
	CNR	33.174(11.202~85.613)	6.254(2.281~40.951)	12.911	61.139	3.084	0.003
Antral portion of the stomach	Signal intensity	100.00(43.170~414.950)	30.360(15.000~112.690)	93.675	428.435	3.133	0.003
	SNR	2.446(2.004~3.485)	2.883(1.744~4.741)	-0.923	0.607	-0.414	0.68
	CNR	20.730(7.669~57.532)	4.984(1.000~14.436)	16.35	57.624	3.6	0.001
Duodenal bulb	Signal intensity	357.740(173.760~1163.670)	70.910(19.010~328.570)	311.565	670.704	5.494	<0.001
	SNR	2.447(1.908~3.221)	3.490(2.251~5.156)	-1.608	0.201	-1.562	0.125
	CNR	95.898(34.723~138.044)	18.293(2.161~60.776)	51.342	105.185	5.839	<0.001
Descending portion of the duodenum	Signal intensity	327.330(98.860~610.010)	60.750(17.740~257.850)	234.756	520.116	5.313	<0.001
	SNR	2.611(2.216~3.261)	3.517(2.286~4.664)	-1.662	-0.33	-3.006	0.004
	CNR	62.878(26.813~117.480)	11.923(2.265~35.517)	39.731	80.168	5.955	<0.001
Horizontal portion of the duodenum	Signal intensity	199.900(62.040~600.470)	47.610(15.950~336.550)	132.549	337.91	4.601	<0.001
	SNR	2.969(2.381~3.858)	2.983(2.255~4.044)	-2.653	0.562	-1.306	0.197
	CNR	33.959(14.767~62.260)	8.721(1.884~33.050)	16.356	45.677	4.249	<0.001
Ascending portion of the duodenum and proximal small intestine	Signal intensity	102.620(43.960~366.420)	27.960(13.630~263.620)	5.159	133.769	2.17	0.035
	SNR	2.714(2.329~3.631)	3.585(2.432~4.775)	-1.219	0.198	-1.448	0.154
	CNR	19.374(8.927~42.036)	4.328(1.084~23.257)	2.459	19.462	2.589	0.013

## Data Availability

All data generated or analyzed during this study are included in this published article.

## LIST OF ABBREVIATIONS

MRCP: Magnetic Resonance Cholangiopancreatography
FRFSE: Fast Recovery Fast Spin Echo
SNR: Signal-to-Noise Ratio
CNR: Contrast-to-Noise Ratio
ICP-AES: Inductively Coupled Plasma-Atomic Emission Spectrometry
ROI: Regions Of Interest
ICC: Intraclass Correlation Coefficients

## STANDARDS OF REPORTING

STROBE guidelines were followed.
